# Novel anthraquinone amide derivatives as potential glyoxalase-I inhibitors

**DOI:** 10.25122/jml-2023-0257

**Published:** 2024-01

**Authors:** Mohammed Al-Akeedi, Manal Najdawi, Qosay Al-Balas, Mohammed Bashar Al-Qazzan, Soha Taher Telfah

**Affiliations:** 1 Department of Applied Pharmaceutical Sciences and Clinical Pharmacy, Faculty of Pharmacy, Isra University, Amman, Jordan; 2 Department of Medicinal Chemistry and Pharmacognosy, Faculty of Pharmacy, Jordan University of Science & Technology, Irbid, Jordan; 3 Department of Pharmaceutical Sciences, Faculty of Pharmacy, Al-Ahliyya Amman University, Amman, Jordan; 4 Department of Pharmaceutical Sciences, Faculty of Pharmacy, Philadelphia University, Amman, Jordan

**Keywords:** Glyoxalase-I, anthraquinone amide derivatives, zinc-binding group, molecular docking, CDOCKER, molecular dynamics, pharmacokinetics

## Abstract

This study aimed to identify novel Glyoxalase-I (Glo-I) inhibitors with potential anticancer properties, focusing on anthraquinone amide-based derivatives. We synthesized a series of these derivatives and conducted in silico docking studies to predict their binding interactions with Glo-I. In vitro assessments were performed to evaluate the anti-Glo-I activity of the synthesized compounds. A comprehensive structure-activity relationship (SAR) analysis identified key features responsible for specific binding affinities of anthraquinone amide-based derivatives to Glo-I. Additionally, a 100 ns molecular dynamics simulation assessed the stability of the most potent compound compared to a co-crystallized ligand. Compound MQ3 demonstrated a remarkable inhibitory effect against Glo-I, with an IC_50_ concentration of 1.45 µM. The inhibitory potency of MQ3 may be attributed to the catechol ring, amide functional group, and anthraquinone moiety, collectively contributing to a strong binding affinity with Glo-I. Anthraquinone amide-based derivatives exhibit substantial potential as Glo-I inhibitors with prospective anticancer activity. The exceptional inhibitory efficacy of compound MQ3 indicates its potential as an effective anticancer agent. These findings underscore the significance of anthraquinone amide-based derivatives as a novel class of compounds for cancer therapy, supporting further research and advancements in targeting the Glo-I enzyme to combat cancer.

## INTRODUCTION

Cancer is a complex and devastating disease characterized by uncontrolled cell growth and metastasis, contributing significantly to global mortality rates [1]. The urgent need for effective therapeutic targets in the form of macromolecules to combat cancer is underscored by the fact that approximately 9.6 million lives were lost to cancer in 2018 alone [2]. One promising target for anticancer drugs is the Glyoxalase system, which plays a critical role in cancer pathology and offers unique opportunities to develop novel antineoplastic medications [3,4]. The Glyoxalase system functions as a detoxification pathway by converting toxic metabolites such as methylglyoxal into non-toxic compounds, particularly D-lactic acid, through a glutathione (GSH)-dependent process [5,6]. The system comprises two main enzymes, Glyoxalase I (Glo-I) and Glyoxalase II (Glo-II). Methylglyoxal is non-enzymatically converted to thiohemiacetal, which serves as the substrate for Glo-I. Glo-I then catalyzes the isomerization of thiohemiacetal into S-D-lactoylglutathione, subsequently hydrolyzed by Glo-II, resulting in the production of non-toxic D-lactic acid and reduced glutathione [5,7-8].

**Figure 1:**
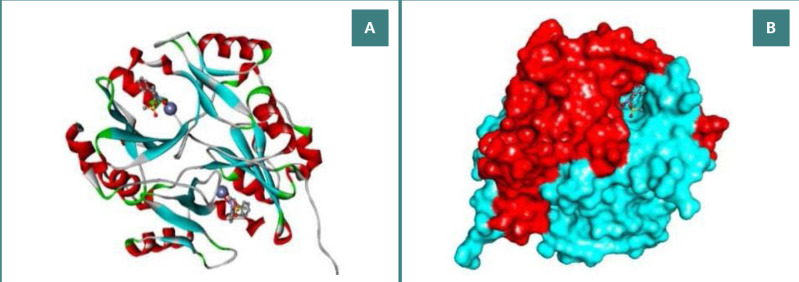
Glo-I crystal structure in humans (PDB code 3W0T). A, Cartoon representation of the human Glo-I structures, with the ligand displayed as balls and sticks and Zn^+2^ ions as gray spheres; B, The human Glo-I displayed surface structure showing the binding sites at the homodimer interface.

In humans, the Glo-I enzyme is a homodimeric mononuclear zinc metalloenzyme with a mass of 42 kDa and monomers of 184 amino acids for each [9]. The dimer interface of Glo-I contains two identical active sites, as illustrated in Figure 1. Glo-I active site comprises 3 distinct areas: a positively charged entrance, a middle area with a zinc atom, and a deep area with a hydrophobic pocket [10]. Understanding the intricate mechanisms underlying the Glyoxalase system and the structural characteristics of Glo-I is essential for designing and developing targeted cancer therapies. By elucidating these mechanisms and structural details, researchers can gain valuable insights into developing effective treatments against cancer. These efforts are crucial for addressing the high mortality rates associated with cancer and improving the prognosis for affected individuals.

The most important area of the active site is the zinc-binding region, which is situated at the bottom of the active site. It involves coordination with three amino acids: Glu99, Gln33, and His126. It is important to recognize that zinc ions commonly have a coordination number of four and a tetrahedral ligand arrangement in protein structures, which allows the zinc atom to form an additional coordinate bond with the inhibitor's zinc-binding group [11]. This characteristic must be considered during the development of Glo-I inhibitors. Glyoxalase I enzyme has a second binding area, a small hydrophobic pocket that can hold up to two aromatic rings. The amino acids Leu 92, Phe 71, Met 179, Leu 160, Leu 69, and Phe 62, which all have a hydrophobic nature, make up this hydrophobic area. In addition, at the entrance of the active site, the enzyme has a highly polar binding region. It is made up of the positively charged side chains of the polar amino acids Arg 122, Arg 37, Lys 150, and Lys 156 [9,12,13]. By investigating potent Glo-I inhibitors in the literature, a series of zinc-binding groups can be extracted. Within this work, a series of suggested compounds were designed based on carbonyl, amide, catechol, and hydroxyl groups as zinc chelating groups in addition to hydrophobic moieties to fill the hydrophobic pocket of the active site. The designed compounds were docked using Discovery Studio 2022 from BioVia, and the highest-ranking compounds were synthesized and tested in vitro to evaluate their inhibition activity against Glo-I. Then, this inhibition capability was confirmed using in silico tools such as pharmacokinetic evaluation and molecular dynamics simulation.

## MATERIAL AND METHODS

### Computational analysis

In total, 44 compounds were drawn using ChemDraw-12 software. These compounds were designed by choosing building blocks with a high potential to coordinate zinc functional groups. A small library of compounds was designed to help us choose compounds to synthesize depending on the docking score (Structure-Based Virtual Screening [SBVS]). This was done to save time and effort in synthesizing compounds with low docking-score.

**Figure 2:**
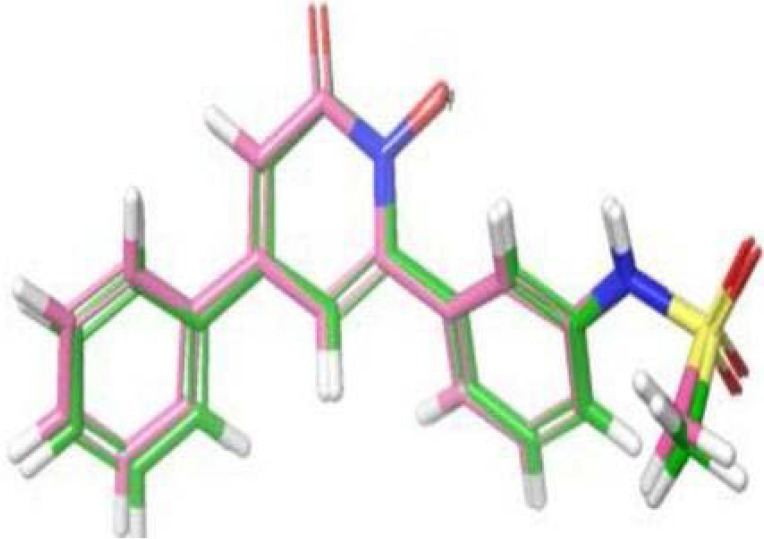
Perfect overlap between the docking pose from the grid (3W0T) and the ligand pose from the co-crystal structure

The compounds were imported into Discovery Studio (DS) for conversion to their 3D structures using the 'Prepare Ligand' protocol. This essential step ensured accurate bond orders, ionization states, and the determination of chemical isomers and tautomers. The Glo-I enzyme representation was chosen from the Protein Data Bank (PDB code: 3W0T), selected for its detailed resolution and the presence of a co-crystallized ligand. The “Prepare Protein” protocol was used to complete the structure by inserting the missing loops, adding missing atoms, and assigning 3D coordinates. All the previous results were issued based on the default parameters of the protocol. Next, the active site was assigned to a radius of 10 A° around the bound ligand, including the zinc atom. To validate the docking process, the co-crystallized ligand was initially redocked and then removed, preparing the protein for docking the designed compound library. The docking process was verified by comparing the ligand pose from docking (under different conditions) with the ligand from the crystal form by performing self-docking to the native co-crystallized ligand through the Root Mean Square Deviation (RMSD) value. Generally, an RMSD value below two angstroms is considered an acceptable overlap. Lower values indicate higher similarity. This criterion enabled us to identify the most accurate docking conditions and ligand poses (Figure 2).

To prepare the crystal structure for molecular docking, the simulation tools within the software package DS (Discovery Studio) were utilized. The initial step involved solvating the crystal structure and performing energetic minimization to optimize its conformation. Subsequently, water molecules were removed, and the co-crystalized ligand was used to define the binding site through the 'Define and Edit Binding Site' tool. Following this, the co-crystalized ligand was eliminated from the structure. A sphere with a radius of 10 Å was created to encompass the defined binding site. The docking of ligands into the active site of Glo-I was performed using two different docking protocols: CHARMM-based docking (CDOCKER) and LibDock. CDOCKER utilizes a protocol based on the CHARMM force field to calculate the binding energy and predict ligand poses within the active site. On the other hand, LibDock is a high-throughput docking algorithm that efficiently explores ligand conformations and placements in the receptor site. Both CDOCKER and LibDock were applied to dock the native and designed ligands into the active site of Glo-I using the crystal structure with the PDB code 3W0T, which has a resolution of 1.35 Å [14]. These docking protocols allowed for the prediction of ligand binding modes and provided insights into the interactions between the ligands and the active site residues of Glo-I.

### Chemistry and synthesis

The necessary chemicals, solvents, and reagents were procured from reputable vendors such as Riedel-de Haen, Sigma-Aldrich, Aldrich, Fluka, Ubichem, Tedia, Merck, and Uniwise Corporation through local subagents. These materials were utilized without undergoing any additional purification steps. The Nuclear Magnetic Resonance (NMR) spectra were recorded using a Bruker 400 MHz-Avance III (400 MHz) spectrometer at the Faculty of Pharmacy, Jordan University of Science and Technology. The chemical shifts in the NMR data are reported in parts per million (ppm) relative to tetramethyl silane (TMS), which served as the internal standard. Deuterated dimethyl sulfoxide (DMSO) was employed as the solvent for sample preparation unless otherwise specified. Fourier Transform Infrared (FTIR) spectroscopy was conducted using a Shimadzu 8400F FT-IR spectrophotometer from Japan at the Faculty of Pharmacy, Isra University. The FTIR analysis involved the utilization of infrared light to scan and analyze the chemical characteristics of the studied materials.

### Synthesis of N-(9, 10-dioxo-9, 10-dihydroanthracen-2-yl) cinnamamide

#### Procedure

**Figure 3:**
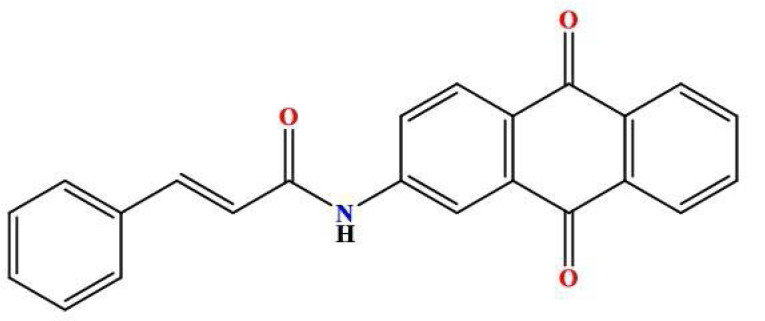
2D structure of the synthesized product MQ4

A mixture of 2-aminoanthraquinone (0.87 g, 1.8 mmol) and cinnamoyl chloride (0.43 g, 3.45 mmol) was prepared in a round-bottom flask equipped with an air condenser. The reaction mixture was heated to 120 °C and maintained under reflux conditions for 18 hours to facilitate the fusion of reactants. Following the reflux step, 1,4-dioxane (15 ml) was introduced into the reaction mixture. The resulting solid mixture was subsequently agitated for an additional 24 hours at ambient temperature. Subsequently, the mixture was permitted to undergo cooling, and the resulting product MQ4 (Figure 3) was extracted through filtration. Ethanol was employed to wash the isolated product, which was subsequently dried. To obtain a purified sample, the product was subjected to crystallization from chloroform, ultimately yielding a yellow solid (1.28 g, 92.94% yield) [15]. The retention factor (Rf) value of the compound was determined to be 0.90 using a chromatographic system consisting of chloroform and methanol in a ratio of 9.8:0.2.

**^1^HNMR:** (400 MHz, DMSO) δ = 7.049 (d, 1H, J = 15.6 Hz, *trans*-CH=CH), 7.094 (t, 2H, aroma), 7.445 (t, 1H, aroma), 7.451 (d, 1H, J = 15.2 Hz, *trans*-CH=CH), 7.695 (d, 2H, aroma), 7.721 (d, 1H, aroma), 7.805 (d, 1H, aroma), 7.942 (s, 1H, aroma), 8.187 (t, 2H, aroma), 8.255 (d, 2H, aroma), 9.116 (s,1H,R2NH).

**^13^CNMR:** (100.61 MHz, DMSO) δ = 117.4, 120.1, 124.2, 126.1 (2C), 127.6 (2C), 128.4, 128.6 (2C), 129.1 (2C), 131.8, 133.25, 134, 134.1, 134.2, 134.4, 139.4, 141.4, 163.7 (N-C=O), 182.2 (2C) ((R2C=O)2) ppm.

**IR:** 3343(CON-H), 2614 (C-H aromatic), 1627, 1664 (C=O ketone), 1690(C=O of amide); GC-MS: m/z = 353.

### Synthesis of (E)-3-(3, 4-dihydroxyphenyl)-N-(9, 10-dioxo-9, 10-dihydroanthracen-2-yl) acrylamide

#### Procedure

**Figure 4:**
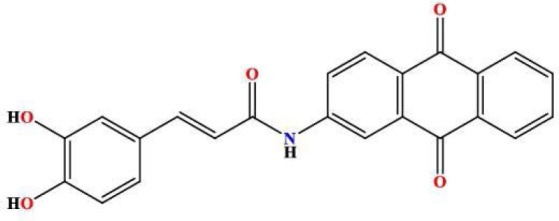
2D structure of the synthesized product MQ3

In a round-bottomed flask, 2-aminoanthraquinone (0.935 g, 1.77 mmol) and caffeic acid chloride (0.43 g, 4.19 mmol) were mixed. The mixture was subjected to an air condensation reaction at 120°C on a hot plate for 24 hours. After condensation, 1,4-Dioxan (15 ml) was incrementally introduced into the flask while stirring at room temperature for an additional 24 hours. Thin layer chromatography analysis confirmed the complete consumption of the starting materials, yielding the desired product MQ3 (Figure 4). An additional spot corresponding to caffeic acid was detected at the baseline. The reaction mixture was gradually cooled using crushed ice for approximately 15 to 20 minutes to facilitate crystallization. The resultant product was isolated via suction filtration, forming a dark-brown solid (1.51 g, 93.55% yield). The Rf value of the compound was determined to be 0.79 using a chromatographic system comprising chloroform and methanol in a 9.8:0.2 ratio.

**^1^HNMR:** (400 MHz, DMSO) δ = 6.672 (d, 1H, aroma), 6.682 (d, 1H, aroma), 6.876 (s, 1H, aroma), 7.156 (d, 1H, *J* = 16.8 Hz, *trans*-CH=CH), 7.336 (d, 1H, *J* = 16.4 Hz, *trans*-CH=CH), 7.567 (d, 1H, aroma), 7.897 (d, 1H, aroma), 7.918 (s, 1H, aroma), 8.095 (t, 2H, aroma), 8.121 (d, 2H, aroma), 9.113 (s,1H,R_2_NH), 9.562 (s,2H,OH);

**^13^CNMR:** (100.61 MHz, DMSO) δ = 115.4, 115.9, 117.1, 118.3, 121.3, 122.1, 122.3 (2C), 126.7, 128.6, 129.2, 132.5 (2C), 135.0 (2C), 135.9, 141.5, 142.5, 145.5, 146.8, 164.9 (N-C=O), 182.5 (2C) ((R_2_C=O)2) ppm.

**IR:** 3336 (aromatic-OH), 1629, 1654 (C=O ketone), 1672 (C=O of amide); GC-MS: m/z = 385.

### Synthesis of N-(9, 10-dioxo-9, 10-dihydroanthracen-1-yl) cinnamamide

#### Procedure

**Figure 5:**
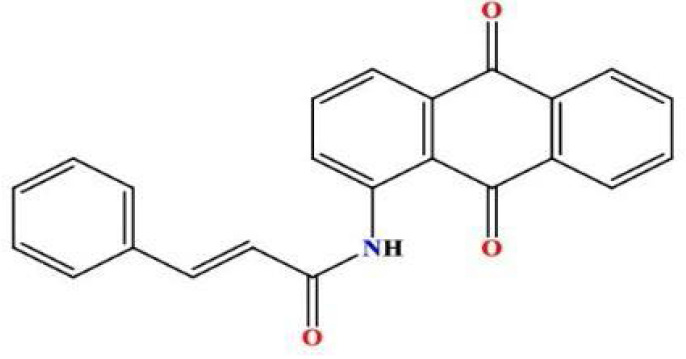
2D structure of the synthesized product MQ5

1-aminoanthraquinone (0.42g, 4.46 mmol) was finely crushed and mixed with cinnamoyl chloride (0.43g, 4.19 mmol). The mixture was refluxed with an air condenser at 120 °C for 18 hours. Then 1, 4-dioxane (15 mL) was added and stirred for 24 hours at room temperature. The separated product, MQ5 (Figure 5), was cooled and filtered, resulting in a pale-yellow powder (0.318 g, 47.83%), Rf: 0.88 (chloroform: methanol, 9.8: 0.2).

**^1^HNMR:** (400 MHz, DMSO) δ = 7.061 (d, 1H, *J* = 16.8 Hz, *trans*-CH=CH), 7.322 (t, 1H, aroma), 7.330 (t, 2H, aroma), 7.531 (d, 1H, *J* = 15.6 Hz, *trans*-CH=CH), 7.758 (d, 2H, aroma), 7.765 (d, 1H, aroma), 7.770 (t, 1H, aroma), 7.845 (d, 1H, aroma), 8.097 (d, 1H, aroma), 8.459 (t, 2H, aroma), 10.872 (s,1H,R_2_NH).

**^13^CNMR:** (100.61 MHz, DMSO) δ = 119.6, 121.3, 123.7, 126.1 (2C), 126.8, 127.7 (2C), 128.3, 128.8 (2C), 132.9, 133.0 (2C), 133.9, 134.0, 134.2, 134.4, 139.3, 141.4, 164.1 (N-C=O), 181.1, 182.2 ((R_2_C=O)_2_) ppm.

**IR:** 3059 (CON-H), 1630, 1667 (C=O ketone), 1692(C=O of amide); GC-MS: m/z = 353.

### Synthesis of (E)-3-(3, 4-dihydroxyphenyl)-N-(9, 10-dioxo-9, 10-dihydroanthracen-1-yl) acrylamide

#### Procedure

**Figure 6:**
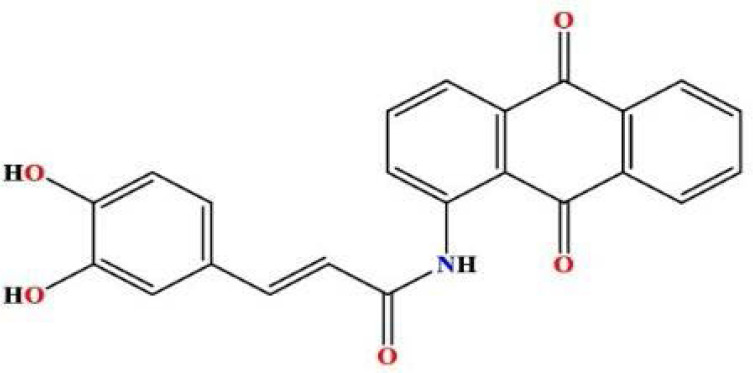
2D structure of the synthesized product MQ1

The reflux process was initiated by combining 1-aminoanthraquinone (0.40 g, 1.8 mmol) and caffeic acid chloride (0.43 g, 3.45 mmol) in the presence of an air condenser. The reaction mixture was then refluxed at 120 °C for 18 hours. Afterward, 15 ml of 1,4-dioxane was added, and the resulting mixture was further agitated for an extra 24 hours at ambient temperature. Upon cooling, the product MQ1 (Figure 6) was isolated via filtration, followed by ethanol washing, drying, and subsequent crystallization from chloroform. These purification steps formed a dark red solid (0.207 g, 29.97% yield). Using a chromatographic system consisting of chloroform and methanol in a ratio of 9.8:0.2, the Rf value of the compound was determined at 0.77.

**^1^HNMR** (400 MHz, DMSO) δ = 6.756 (d, 1H, aroma), 6.792 (d, 1H, aroma), 6.810 (s, 1H, aroma), 7.095 (d, 1H, *J* = 16.4 Hz, *trans*-CH=CH), 7.427 (d, 1H, *J* = 15.2 Hz, *trans*-CH=CH), 7.609 (d, 1H, aroma), 7.710 (t, 1H, aroma), 7.845 (d, 1H, aroma), 8.097 (d, 2H, aroma), 8.486 (t, 2H, aroma), 9.556 (s,2H,OH), 10.872 (s,1H,R_2_NH);

**^13^CNMR** (100.61 MHz, DMSO) δ = 112.2, 114.0, 116.3, 118.7, 121.3, 122.7, 126.0 (2C), 128.1, 129.6 (2C), 130.2, 131.1 (2C), 132.1, 134.4, 139.6, 141.3, 144.1, 145.3, 167.6 (N-C=O), 180.0, 183.2 ((R_2_C=O)_2_) ppm.

**IR:** 3305 (aromatic-OH), 1628, 1668 (C=O ketone), 1683(C=O of amide); GC-MS: m/z = 385.

### In vitro Enzyme Inhibition Assay

Glo-I inhibitory action was evaluated following the methodology described by Al-Balas *et al*. [16,17]. Recombinant human Glo-I (rhGlo-I) was procured from R&D Systems Corporation and thawed in sterile deionized water at a precise concentration of 0.5 mg/ml before being reconstituted. The reconstituted rhGlo-I was stored at -70 °C until further use. Dimethyl sulfoxide (DMSO) was used to dissolve the test compounds to produce stock solutions with a 10 mM concentration. The assay buffer required for the experiment was made by mixing 0.1 M sodium phosphate monobasic solution with 0.1 M sodium phosphate dibasic solution, resulting in a pH range of 7.0–7.2. The reduced glutathione and methylglyoxal solutions were combined with a sufficient 0.1 M sodium phosphate assay buffer volume to create the substrate combination. In a cuvette, the assay buffer, substrate solution, and Glo-I enzyme were combined with the synthesized products at the required concentration. All synthesized compounds were subjected to a triplicate bioassay, and the reactions were monitored for 200 seconds at a temperature of 25 °C. The maximum absorbance (γmax) was recorded at 240 nm.

### In-silico pharmacokinetics prediction for the best-docked structure

To assess the drug-likeness properties of the top-performing docking structure (designated as MQ3), we conducted an in-silico pharmacokinetics prediction study utilizing the ADMET Descriptors protocol within the Biovia Discovery Studio. This study evaluated the toxicological endpoints through a Quantitative Structure Toxicity Relationship (QSTR) approach. Specifically, we examined the Ames mutagen prediction, Ames probability, Ames enrichment, and weight of evidence, collectively forming a comprehensive toxicity profile for the investigated compounds.

### Molecular Dynamics evaluation

To explore the binding affinities of MQ3, which demonstrated the highest binding affinity to the active site of the Glo-I protein, a rigorous evaluation was performed through a 100 nanosecond Molecular Dynamics (MD) simulation, followed by a comparative analysis with the crystallographically native ligand. For this simulation, GROMACS-2022 was employed, with particular attention to the preparation of the Glo-I protein topology using the CHARMM36 force field. Likewise, the ligand topology was meticulously generated using the CHARMM force field through the General Force Field (CGenFF) server. The complexes were then immersed in a dodecahedral box under 10 Å boundary conditions, and ionization was executed employing the steepest descent minimization algorithm to achieve a neutral charge. Subsequently, the systems underwent energy minimization employing the same algorithm, employing a 10.0 kJ/mol cutoff. Following this stage, both the NVT (constant number of particles, volume, and temperature) and NPT (constant number of particles, pressure, and temperature) equilibration processes were conducted for 10 picoseconds, employing a time step of 2 femtoseconds. This preparatory phase was followed by a comprehensive 100-nanosecond MD simulation of the resulting complexes. In the post-simulation analysis, the Molecular Mechanics Poisson Boltzmann Surface Area (MM/PBSA) approach was used to compute the binding free energies and the solvent-accessible surface area (SASA) model to calculate non-polar solvation energies.

## RESULTS

### Compound Design Criteria

The design process employed in this study adheres to specific criteria, including incorporating a zinc-chelating moiety, the presence of a hydrophobic moiety, the availability of suitable building blocks, and the practical feasibility of synthesizing the targeted compounds. The 'zinc binding groups' selected for consideration include catechol, carbonyl, amide, and enol, while a variety of aromatic hydrophobic groups were chosen, including phenyl, furan, naphthol, and indole. Including a hydrophobic moiety is of paramount importance, as it occupies a hydrophobic pocket adjacent to the zinc atom within the active site. In pursuit of these objectives, forty-four compounds were proposed as potential inhibitors of Glo-I. These compounds were sketched using ChemDraw software and converted into their respective three-dimensional (3D) structures using Discovery Studio. Subsequently, these suggested compounds underwent preparation through the 'Prepare Ligand' protocol, with all parameters maintained at default settings, permitting the protocol to mimic ionization similar to a biological system and to generate isomers and tautomers. The final cohort of ligands, ready for docking within the active site of Glo-I, amounted to 134, including two compounds selected as 'reference' ligands based on their binding affinity compared to the crystal structure and the native co-crystallized ligand (N-hydroxypyridone derivative). Table 1 overviews the synthesized compounds and their respective IC_50_ values. Specifically, two compounds, the hydroxamic acid derivative (compound 18) and the flavone derivative (Baicalein, compound 19), were chosen as reference compounds to validate the docking process and as a benchmark for evaluating the expected activity of the suggested compounds. Furthermore, the reported IC_50_ values from the literature for these reference compounds were included in Table 1 for comprehensive reference.

**Table 1:** The structures of the synthesized compounds with their IC_50_ values, in addition to the two reference compounds for a computational run

Compound name	Structure	LibDock Score	CDOCKER Score	IC_50_
N-hydroxypyridone derivative (co-crystallized ligand)	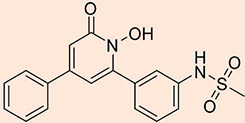	128.922	46.4319	Not reported
Hydroxamic acid derivative	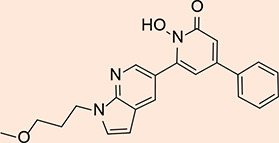	135.477	56.0727	11 nM
Baicalein	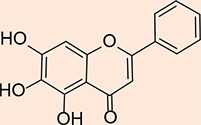	116.167	44.8419	11 µM
MQ1	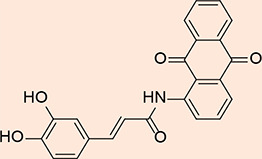	135.322	54.0697	27.92 µM
MQ5	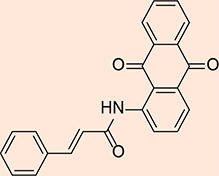	123.138	49.5768	107.3 µM
MQ3	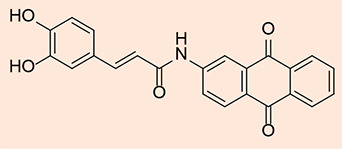	135.093	52.7683	1.45 µM
MQ4	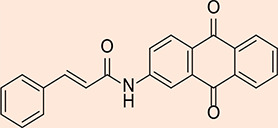	120.185	50.1358	14.45 µM

### Molecular docking

**Figure 7:**
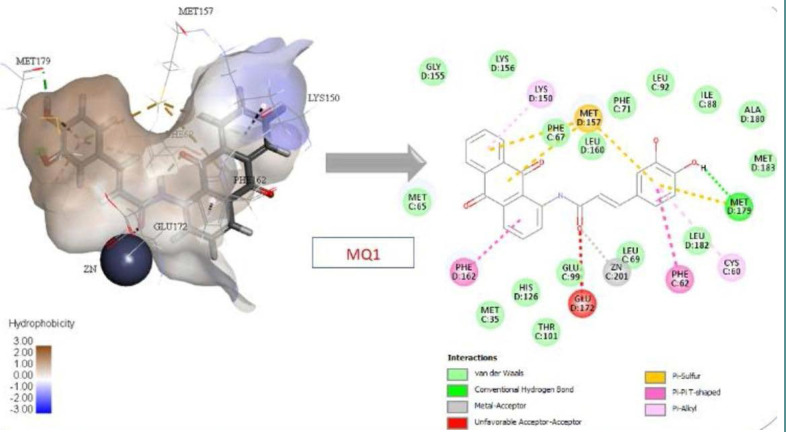
Compound MQ1 interactions with Glo-I active site

The 'Prepare Protein' protocol was used, all missing loops and missing atoms were inserted, and the 3D coordinates were assigned to the Glo-I crystal structure. All the previous results were issued based on the default parameters of the protocol. Next, the active site was assigned to a radius of 10 Å around the bound ligand, including the zinc atom. After removing the co-crystallized ligand, the protein was primed for docking. Two docking protocols available in Discovery Studio 2022 were used: CDocker and LibDock. CDocker protocol was used to dock the candidate compounds. This software is a validated and reliable tool offered by BioVia Company with acceptable accuracy. Each compound produces 10 poses, and their scores are presented as CDocker interaction energy. LibDock is an efficient algorithm for high-throughput ligand docking into an active receptor site. During the process, ligand conformations are aligned to apolar and polar receptor interaction sites, known as hotspots, and the algorithm identifies and reports the best-scoring poses. The best compound with the highest score was compound MQ1 in both docking protocols (CDocker: 54.06; LibDock: 135.3). This compound has a structural complementarity with the active site. Its catechol moiety is within the hydrophobic pocket, while one hydroxyl performs hydrogen bonds with Met179. Additionally, the compound's amide group chelates the zinc atom, while its anthraquinone ring engages in π-π interactions with Phe162 at the active site's positively ionized entrance. The amide group of the compound chelates the zinc atom, and its anthraquinone ring engages in π-π interactions with Phe162 at the active site's entrance (Figure 7).

**Figure 8:**
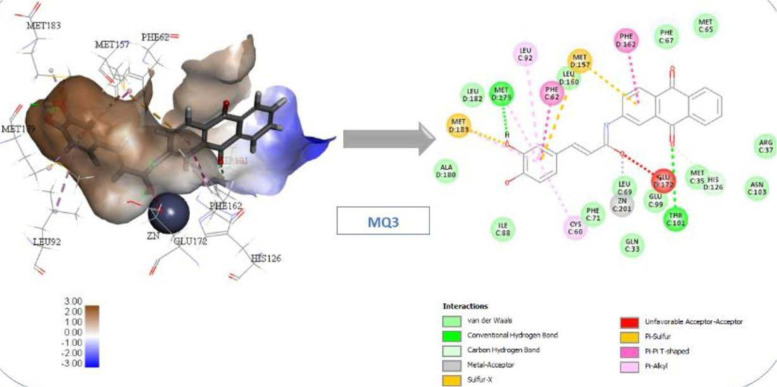
Compound MQ3 interactions with Glo-I active site

All the suggested compounds, including native and reference molecules (a total of 47 compounds), were docked using CDocker and LibDock, and the two compounds with the highest scores were MQ1 and MQ3. These results were anticipated, as both compounds contain an amide moiety, which is recognized for its ability to chelate zinc and a catechol moiety that aligns well with the amino acids from the active site. Furthermore, the anthraquinone moiety occupies the entrance of the active site. These two compounds were synthesized and tested against Glo-I to investigate their activity. Interestingly, they were active in vitro against Glo-I and scored an IC_50_ of 27.92 µM and 1.45 µM, respectively. To verify the importance of the catechol moiety within these compounds, their counterparts (MQ5 and MQ4), which lack the catechol moiety, were synthesized and tested against the Glo-I enzyme. As expected, their IC_50s_ were more than 50 µM and 14.5 µM, respectively. Considering the limitations in this project related to the availability of the starting materials and the interrupted chain supplies of the ordered chemicals, we were able to synthesize only four compounds and perform full structural characterization. The synthesized compounds were validated, and the activity was determined in vitro. Comparing the synthesized compounds to our reference ones, the presence of hydroxamic moiety has imparted compound (1) superiority as this functional group is classified as a strong zinc chelator and performs bidentate strategic binding. On the other hand, compound (2) Baicalein (reference 2) scored less docking value than our suggested compounds (MQ1 and MQ3) and less in vitro activity than that of MQ3, a good sign of the accuracy of the docking software of predicting biological activity. The docking results showed that compounds MQ1 and MQ3 scored high with comparable values to the reference hydroxamic acid compound (1) (Table 1). MQ4 and MQ5 were selected because they lack the catechol moiety present in MQ1 and MQ3. This was designed to expose the importance of catechol moiety in binding with the active site and its effect on the inhibitory activity against Glo-I. The returned values of MQ4 and MQ5 were lower than reference compound 1 and lower than their counterparts, MQ1 and MQ3. According to the docking scores in Table 1, the CDocker interaction energy of compound MQ1 was higher than compound MQ3. The binding interactions of compound MQ3 with the active site of the Glo-I enzyme are shown in Figure 8.

**Figure 9:**
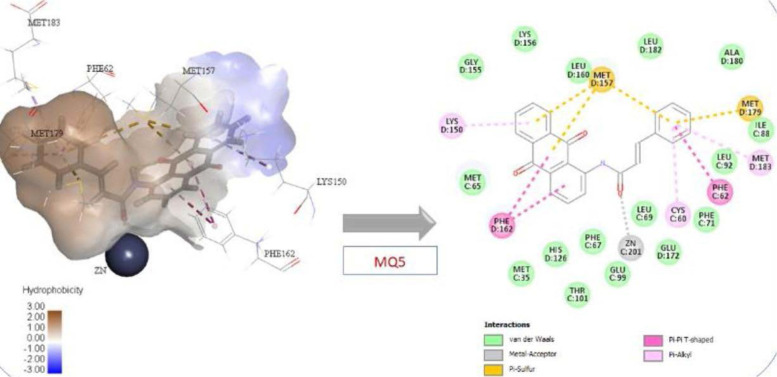
Compound MQ5 interactions with Glo-I active site

These interactions were π-π stacking between the aromatic rings of anthraquinone with Phe162 and H-bond with Thr101. The catechol ring of caffeic moiety made another π-π stacking with Phe62 in addition to pi-sulfur interaction with Met157. Another important interaction by catechol moiety was the hydrogen bond with Met179 and π-sulfur with Cys60. The zinc coordination was observed via the carbonyl of the Amide Bridge as a metal acceptor. As indicated in Table 1, Compound MQ5 scores were 49.5 (CDOCKER) and 123.1 (LibDock). The interactions between compound MQ5 and the active site of Glo-I enzyme are displayed in Figure 9. π-π stacking interaction was observed between anthraquinone moiety and Phe162. The benzene ring of cinnamic acid moiety formed two interactions: π-π stacking with Phe62 and π-sulfur interaction with Met157. The zinc atom was coordinated via the carbonyl group of the Amide Bridge. Very important interactions were missing due to the lack of the catechol moiety, which contributed to the lower score of compound MQ5 compared to its catechol counterpart. In addition, the lack of catechol moiety was detected in the in vitro results, which resulted in substantial activity loss.

### In-silico pharmacokinetics prediction for the best-docked structure

ADMET profile of MQ3 included the comprehensive evaluation of critical physicochemical characteristics, including lipophilicity (clog P), polar surface area, molecular weight (MW), aqueous solubility (log S), as well as its interactions with CYP2D6, human intestinal absorption (HIA), plasma protein binding (PPB), its capacity to cross the blood-brain barrier (BBB), and potential hepatotoxicity. A concise summary of the pharmacokinetic profile of MQ3, which highlights its noteworthy attributes, can be found in Table 2. Notably, it demonstrates a commendable level of solubility, classified as level 2, and scores 4 on the BBB penetration scale, indicating a robust ability to permeate the blood-brain barrier. Additionally, its Alog *P* value, signifying lipophilicity, stands at 3.89, aligning with the Lipinski Rule of Five, an important criterion for assessing oral bioavailability. The predicted human intestinal absorption value of 3.54631 corresponds to a level 1 rating, signifying favorable absorption. The CYP2D6 probability assessment yielded a value of -6.27, suggesting that MQ3 is unlikely to function as an inhibitor of the CYP2D6 enzyme, thus avoiding interference with its metabolic processes. Lastly, toxicity assessment using the TOPKAT tool in Biovia Discovery Studio revealed hepatotoxicity and Ames prediction values below 7, indicating a non-mutagenic nature. This collectively establishes a robust safety profile for MQ3.

**Table 2:** Pharmacokinetics profile of MQ3

BBB	Alog P	Solubility level	HIA	PPB	CYP2D6	Hepatotoxicity	Ames-Prediction
4	3.89	2	1	3.54631	-6.27	-3.6	NM

### Molecular Dynamics evaluation

**Figure 10:**
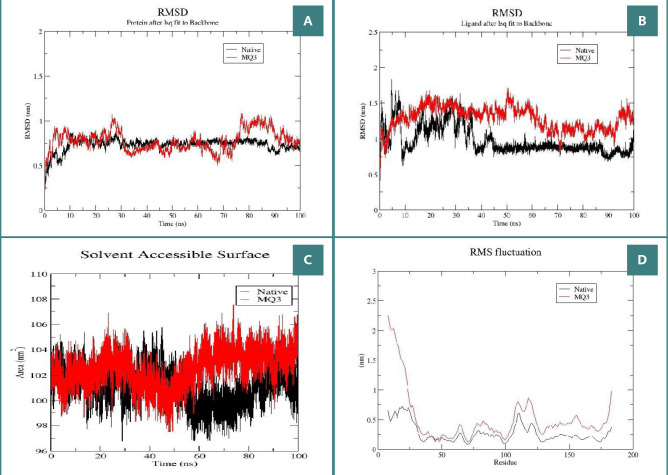
Structural dynamics of vorinostat (red) and Native (black) bound to Glo-I. A, Backbone RMSD punicalagin RMSD; B, SASA values; C, RMSF; D, calculated during the 100 ns of MD trajectories.

To validate the stability of MQ3 binding to the Glo-I target and to make a comparative assessment with the native ligand, a rigorous examination was conducted by subjecting the complexes to a 100 nanosecond MD simulation, employing GROMACS-2022. This simulation facilitated an in-depth analysis of the conformational changes exhibited by the protein-ligand complexes, serving to assess the overall stability of the simulated systems. Various parameters, including RMSD for the protein backbone and the ligand, radius of gyration, and solvent-accessible surface area, were meticulously scrutinized for the 100 nanosecond MD simulation (Figure 10). The RMSD plot for the Glo-I protein backbone (Figure 10A) reveals that the RMSD values for MQ3 (in red) exhibit stability when compared to those of the native ligand (in black). RMSD curves demonstrated fluctuations around 0.75 nanometers, a range considered acceptable. Notably, after approximately 10 nanoseconds into the simulation, both protein backbones attained a stable conformation characterized by minimal fluctuation (less than 0.1 nanometers). Similarly, the ligand RMSD plot (Figure 10B) demonstrates that after about 35 nanoseconds, MQ3 and the native ligand exhibited stable binding affinity with low oscillation, maintaining values around 0.5 nanometers. To further confirm this binding stability, we examined the average solvent-accessible surface area (SASA) values of the protein backbone. Both complexes demonstrated non-polar solvation energies with average SASA values of approximately 102 square nanometers, with fluctuations ranging between 98 and 106 square nanometers throughout the entire simulation. This range falls within the acceptable bounds, affirming the high stability of the complexes (Figure 10C). A deeper analysis of the binding stability of our proposed candidate involved a detailed examination of the root-mean-square fluctuation (RMSF) for the same amino acids. As illustrated in the RMSF plot (Figure 10D), a consistent pattern was observed for both ligands, suggesting that MQ3 bound to the same pocket with an affinity similar to those observed in in vitro experiments.

## DISCUSSION

### Chemistry

**Figure 11:**
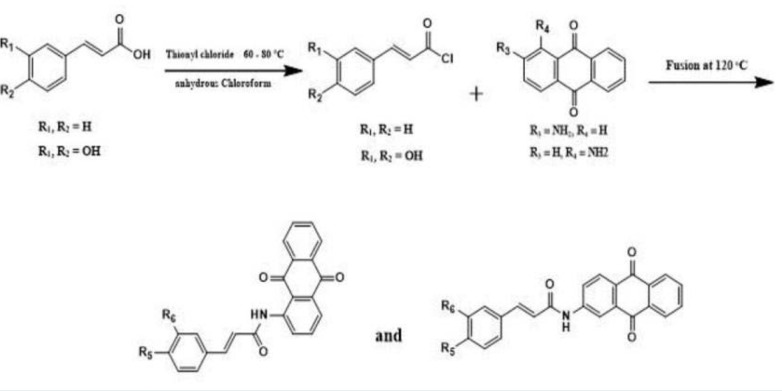
Scheme 1 for the synthesis of the targeted compounds

The assigned compounds, namely MQ1, MQ3, MQ4, and MQ5, were synthesized according to Scheme 1, as depicted in Figure 11.

The synthesis procedure, as outlined in another study [15], was adopted to prepare these compounds. Initially, the carboxylic acid functional groups within the starting materials were transformed into acyl chlorides via refluxing with thionyl chloride in an anhydrous solvent, such as chloroform. This conversion took place within a temperature range of 60 to 80 °C.

Subsequently, the resulting acyl chloride compounds underwent a fusion reaction with anthraquinone derivatives at elevated temperatures. The fusion reaction was conducted under reflux conditions at 120 °C, with a duration of 18 hours, and an air condenser was employed. Following the fusion process, 1,4-dioxane was introduced into the reaction mixture, which was agitated for an additional 24 hours at room temperature to facilitate subsequent reactions and transformations. The desired compounds were obtained through filtration and subsequent recrystallization steps. The synthesized compounds underwent characterization using NMR and IR techniques, confirming the anticipated positions and spectral peaks. Notably, the 1H-NMR spectra revealed singlet peaks at approximately δ ≈ 180 and 190 ppm, corresponding to the two carbonyl groups within the anthraquinone moiety, while the amide carbonyl peak was observed at approximately 160 ppm. A diverse range of functional groups were incorporated into the synthesized compounds, including polar moieties such as hydroxyl groups, the ketone moiety of anthraquinone, and a secondary amide bridge. These functional groups were selected to facilitate interactions with the target protein, either through hydrogen bonding or chelation with the zinc ion of the enzyme. Additionally, hydrophobic moieties such as the benzene ring of cinnamic acid and aromatic rings of anthraquinone were incorporated to enable π-π interactions with amino acid residues of the target protein. The selection of these specific interactions was guided by the positioning at the active site of the target protein, thus enhancing the inhibitory activity of the compounds.

### Glo-I enzyme inhibition bioassay

**Figure 12:**
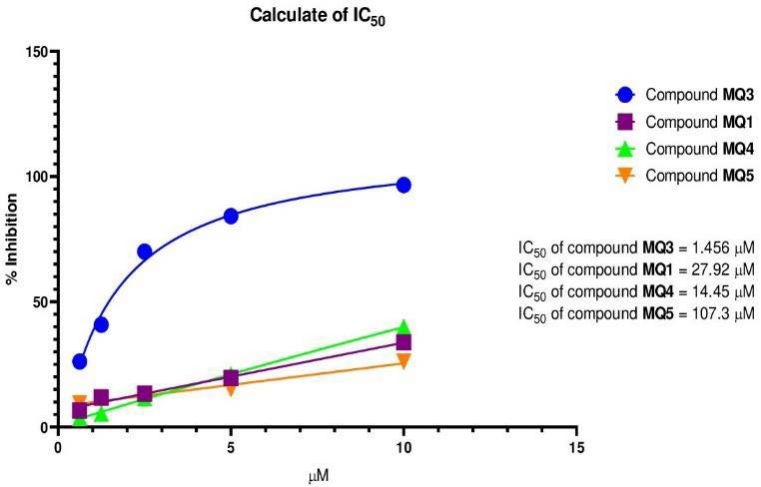
The Glo-1 inhibition activity IC_50_ of synthesized compounds

In this study, the inhibitory activity of all synthesized compounds against the Glo-I enzyme was evaluated in vitro using recombinant human Glo-I obtained from R&D Systems Corporation. The methodology previously described was employed, which involved measuring the inhibition of Glo-I activity and substrate hydrolysis using a spectrophotometer at 240 nm [16,17]. The results were expressed as the percentage of inhibition, and it was observed that the inhibitory activity of the synthesized compounds increased significantly with increasing concentrations (*P* <0.05). Among the synthesized compounds, compound MQ3 exhibited the highest potency with an average inhibitory activity of 96.63% at a concentration of 10 M (Table 3). The activity profile of the tested compounds against Glo-I is depicted in Figure 12. Compound MQ5 displayed an IC_50_ value of 107.3 µM, compound MQ1 had an IC_50_ value of 27.92 µM, compound MQ4 exhibited an IC_50_ value of 14.45 µM, and compound MQ3 demonstrated the most potent inhibitory activity with an IC_50_ value of 1.45 µM.

**Table 3:** Percent of inhibition of Glo-I enzyme activity of the synthesized compounds

Compound MQ3							
**Concentration µM**	**% Inhibition**					**R squared**	**Calculated IC_50_**
10	97.48993	93.79939		98.59585		0.9859	1.456 µM
5	82.31771	84.90233		85.43665			
2.5	69.55614	70.04076		70.55023			
1.25	39.19926	42.52945		40.76495			
0.625	27.46906	28.33888		22.6726			
**Compound MQ1**							
**Concentration µM**	**% Inhibition**					**R squared**	**Calculated IC_50_**
10	33.35046		34.53021		33.45559	0.9474	27.92 µM
5	18.76139		20.11634		19.92945		
2.5	11.81143		13.43503		14.70822		
1.25	12.02168		11.44933		12.03336		
0.625	4.207354		6.928935		8.657665		
**Compound MQ4**							
**Concentration µM**	**% Inhibition**					**R squared**	**Calculated IC_50_**
10	40.44951		41.45382		37.84887	0.9833	14.45 µM
5	19.35893		20.18353		23.18589		
2.5	10.81698		12.29702		11.67329		
1.25	5.034252		3.871363		7.391746		
0.625	6.207713		1.894452		2.93048		
**Compound MQ5**							
**Concentration µM**	**% Inhibition**					**R squared**	**Calculated IC_50_**
10	21.97335		29.00198		27.35604	0.7606	107 µM
5	11.55275		12.80945		20.99468		
2.5	14.31082		12.23114		12.25339		
1.25	10.20708		9.973531		12.33124		
0.625	8.816922		8.550012		10.84099		

### Correlation between the in silico and in vitro results

Despite the prediction that compound MQ1 would be the most potent ligand based on docking results (Table 1), the in vitro inhibition assay revealed that compound MQ3 exhibited the highest effectiveness against the Glo-I enzyme (Figure 8). This outcome was unexpected, considering our previous knowledge that the linearity of the designed compound greatly influences its inhibitory activity. The limitations of molecular modeling in accurately replicating protein dynamics and the influence of the native ligand co-crystallized in the active site may have contributed to this disparity between the docking predictions and experimental results. Our biological assay validated the hypothesis, demonstrating that the linear compound MQ3 was the optimal inhibitor with an IC_50_ value of 1.45 µM. In contrast, the nonlinear compound MQ1, despite containing a catechol moiety, had a much higher IC_50_ value of 27.9 µM. This trend was also observed for the non-catechol compounds, with the catechol derivatives displaying lower IC_50_ values compared to their non-catechol analogs. The consistency between the docking predictions and the experimental data further supports the notion that the presence of the catechol group enhances inhibitory activity.

## CONCLUSION

This study involved the synthesis and assessment of several novel catechol derivatives, specifically MQ1, MQ3, MQ4, and MQ5, for their inhibitory efficacy against the Glo-I enzyme. Among these compounds, MQ3 exhibited remarkable potency, demonstrating an IC_50_ value of 1.45 µM. The exceptional activity observed in MQ3 can be attributed to the incorporation of a zinc-chelating moiety (amide) and a catechol moiety, which collectively enable precise positioning and interactions within the active site of the enzyme. Furthermore, the introduction of anthraquinone in the compound structure played a pivotal role in enhancing inhibitory activity. It facilitated significant π-π stacking interactions at the entrance of the active site, further augmenting the inhibitory potential. Additionally, we noted that the linear conformation of the designed compounds significantly influenced their activity. Linear derivatives exhibited superior inhibitory performance when compared to their non-linear counterparts. This observation underscores the importance of accommodating the unique requirements and characteristics of the active site, which favors linear conformations for optimal ligand binding and inhibition. In summary, our findings underscore the promising inhibitory capabilities of the synthesized catechol derivatives against the Glo-I enzyme. The presence of a zinc-chelating moiety, a catechol moiety, and the inclusion of anthraquinone collectively contribute to their potent activity. The preference for linear compounds further highlights the significance of comprehending the nature of the active site for effective ligand design.
